# Anatomical changes of *Tenebrio molitor* and *Tribolium castaneum* during complete metamorphosis

**DOI:** 10.1007/s00441-024-03877-8

**Published:** 2024-02-27

**Authors:** Maria Luigia Vommaro, Sandro Donato, Simone Caputo, Raffaele G. Agostino, Aurora Montali, Gianluca Tettamanti, Anita Giglio

**Affiliations:** 1 University of Calabria, Department of Biology, Ecology and Earth Science, Rende, Italy; 2 University of Calabria, Department of Physics and STAR research infrastructure, Rende, Italy; 3 Istituto Nazionale di Fisica Nucleare, Division of Frascati, Rome, Italy; 4 University of Calabria, Department of Environmental Engineering, Rende, Italy; 5 University of Insubria, Department of Biotechnology and Life Sciences, Varese, Italy; 6 Interuniversity Center for Studies on Bioinspired Agro-environmental Technology (BAT Center), University of Napoli Federico II, Portici, Italy

**Keywords:** Insect development, Gonad, Midgut, Moult, Organ rendering, Pupa

## Abstract

In holometabolous insects, extensive reorganisation of tissues and cells occurs at the pupal stage. The remodelling of the external exoskeleton and internal organs that intervenes during metamorphosis has been traditionally studied in many insect species based on histological or ultrastructural methods. This study demonstrates the use of synchrotron X-ray phase-contrast micro-computed tomography as a powerful, non-destructive tool for in situ morphological observation of anatomical structures at the pupal stage in two Tenebrionid beetles, i.e. *Tribolium castaneum* and *Tenebrio molitor*, known as important pests, as well as emerging and promising models in experimental biology. Virtual sections and three-dimensional reconstructions were performed on both males and females at early, intermediate, and late pupal stage. The dataset allowed us to observe the remodelling of the gut and nervous system as well as the shaping of the female and male reproductive system at different pupal ages in both mealworm and red flour beetles. Moreover, we observed that the timing and duration pattern of organ development varied between the species analysed, likely related to the species-specific adaptations of the pre-imaginal stages to environmental conditions, which ultimately affect their life cycle. This research provides new knowledge on the morphological modifications that occur during the pupal stage of holometabolous insects and provides a baseline set of information on beetle metamorphosis that may support future research in forensics, physiology, and ecology as well as an image atlas for educational purposes.

**Supplementary Information:**

The online version contains supplementary material available at 10.1007/s00441-024-03877-8.

## Introduction

Complete metamorphosis is one of the main developmental innovations that has likely contributed to shape the great biodiversity of holometabolous insects (Jindra 2019). The evolutionary and adaptive value of the transition from larva to adult, that takes place through the nonfeeding, quiescent pupal stage, has been largely discussed to play a fundamental role in reducing the intraspecific competition between the juvenile and adult form and overcoming adverse seasonal environmental conditions (Rolff et al. 2019). The regulation of the transition from larval to pupal stage and from pupa to adult has been studied in detail, focusing on the endocrine control of ecdysteroid and juvenile hormone (Pan et al. 2021; Truman and Riddiford 2001) that act through a conserved genetic circuit (Martín-Vega et al. 2021; Truman 2019; White et al. 1999). In addition, extensive tissue remodelling occurs at pupal phase and involves the removal of unnecessary or obsolete larval organs, mainly through autophagic and apoptotic cell death (Lee and Park 2021; Tettamanti et al. 2007; Tettamanti and Casartelli 2019), resulting in their replacement with new adult tissues by stem cell proliferation (Caccia et al. 2019; Franzetti et al. 2015).

The external structure of the pupal stage has been extensively described in endopterygotes and the obtect and exarate general morphological types were recognized, depending on whether their appendages are closely appressed or not to the body (Gillott 2005; Gullan and Cranston 2014; Sehnal 1985). However, the internal morphology of the pupa has received limited attention because of the time-consuming processing procedures and the considerable number of samples required for histological analyses to obtain robust results. Studies to date have been mainly focused on dipteran such as *Drosophila melanogaster* (Bainbridge and Bownes 1981) and blowflies of the *Calliphora* genus (Bowen et al. 1996; Brown and Harvey 2014; Crossley 1965; Hall et al. 2017; Martín-Vega et al. 2021; Nur et al. 2019), while only a few species from other holometabolous orders have been investigated in detail. These include the lepidopteran *Vanessa cardui* (Lowe et al. 2013), mecopteran *Panorpa vulgaris* (Saltin et al. 2016), neuropteran *Chrysopa pallens* (Zhao et al. 2020), hymenopteran *Megachile rotundata* (Helm et al. 2018) and coleopteran *Trypoxylus dichotomus* (Ikegami et al. 2023).

The red flour beetle, *Tribolium castaneum* Herbst 1797, and the mealworm beetle, *Tenebrio molitor* Linneus 1758, (Coleoptera, Tenebrionidae) are the most studied human commensals associated with stored grain, flour, or other cereal-based food products (Atta et al. 2020; Rumbos et al. 2020). For this reason, the research to date has primarily focused on their biological cycle, which, for the red flour beetle takes approximately 4–6 weeks and the pupal stage 6–8 days (Chandrasekar and Palli 2011; Chaubey 2023), depending on the temperature and food supply (Skourti et al. 2022). In contrast, the entire developmental process of *T. molitor* lasts 10–12 weeks, including 14 to 18 larval stages and 7–8 days of pupal stage at 28 °C (Arbab 2019; Chandrasekar and Palli 2011; Connat et al. 1991; Morales-Ramos et al. 2015; Park et al. 2014; Yu et al. 2021).

Both beetles are easy to rear under controlled conditions, and their genome have been sequenced in recent years (Kaur et al. 2023; Oppert et al. 2023; Richards et al. 2008). For these reasons, they are useful model species (Brai et al. 2023; Brown et al. 2009; Campbell et al. 2022) for genetic (Suzuki et al. 2008), physiological, immunological (Abdel-Latief and Hoffmann 2014; Park et al. 2014; Vigneron et al. 2019; Vommaro et al. 2021, b), developmental, behavioural (Pai and Bernasconi 2008) and evolutionary (Pointer et al. 2021) studies. In addition, both species are used to address the effects of pesticides (Kostaropoulos et al. 2001; Pedersen et al. 2020), applied to control populations infesting food commodities (Arthur et al. 2019; Ntalli et al. 2021; Tungjitwitayakul et al. 2022), including insect resistance (Campbell et al. 2022; Rösner et al. 2020) and in terms of ecotoxicological effects (Naccarato et al. 2023). Despite the great interest towards these species, the morphology of *T. castaneum* has been scarcely studied (Dönitz et al. 2013; Vommaro et al. 2023a; Zohry and El-Sayed 2019) and the pupal stage has been completely neglected. In *T. molitor*, previous studies have only covered the X-ray microtomographic analysis of the tracheal system (Iwan et al. 2015; Raś et al. 2018) during metamorphosis and the visual structure in the adult (Giglio et al. 2022).

The present study aimed at using the synchrotron X-ray phase-contrast micro-computer tomography to describe the in situ anatomical modifications of pupal stage in both females and males of *T. molitor* and *T. castaneum*. The analyses of virtual body dissections and three-dimensional organ reconstructions help fill in the lack of information regarding the anatomical changes that occur during coleopteran metamorphosis and facilitate future comparative studies, and increase the knowledge of the development of two well-known species, as pests of stored products and emerging models in experimental biology.

## Materials and methods

### Insect rearing, sampling and fixation

Pupae of *T. molitor* were obtained from a laboratory stock population maintained at the Morphofunctional Entomology Laboratory, Dept. of Biology, Ecology and Earth Science, University of Calabria. Mealworm beetle larvae were reared at a relative humidity (RH) of 60% under a natural photoperiod and room temperature (24 ± 2 °C), providing ad libitum diet composed of organic wheatmeal and fruit.

Specimens of *T. castaneum* were obtained from a colony maintained at the Dept. of Biotechnology and Life Sciences, University of Insubria. The beetles were fed with wheat flour supplemented with 5% (w/w) Brewer’s yeast and reared at 30 ± 5 °C, 70 ± 5% RH and a 12:12 h light:dark cycle.

For morphological analyses, 1-, 5- and 8-day-old pupae of *T. molitor* and 1-, 3- and 5-day-old *T. castaneum* pupae, corresponding to the early, intermediate, and late pupal stages, were collected. The time points for the analysis of the anatomical changes in the pupae were chosen based on shifts in cuticle pigmentation of eyes, mandibles, legs, and the body wall from the newly moulted pupal stage to the later pupal-adult moult. Specimens were anesthetised in a cold chamber at 4 °C for three minutes and prepared as indicated in Donato et al. (2021). Briefly, insects were fixed in 2.5% glutaraldehyde and 1% paraformaldehyde in 0.1 M phosphate buffer, pH 7.4 (PBS) (Electron Microscopy Sciences, United States) overnight at 4 °C, washed with PBS, and dehydrated in a graded ethanol series. Each sample was housed in a 1.5 ml plastic microtube containing ethanol for analysis.

### Phase contrast micro-computed tomography (PhC micro-CT) data acquisition

The SYRMEP (SYnchrotron Radiation for MEdical Physics) (Dullin et al. 2021) beamline at the Elettra Synchrotron Facility in Trieste (Italy) was used for tomographic data acquisition. One of the storage rings bending magnets generated X-rays in the energy range from 8.5 to 40 keV. Each sample was illuminated with a polychromatic radiation, collecting N = 1800 equal-angle projections over 180°. Continuous rotation mode was used for detection with a water-cooled Hamamatsu sCMOS detector, which was optically coupled to a GGG (Gd3Ga5O12:Eu) scintillator (with a sensitive layer thickness of 45 µm), using a series of optical lenses to set different magnification levels (Donato et al. 2022). The detector had a native pixel size of 6.5 µm × 6.5 µm, with the sensor comprising an area of 2048 × 2048 pixels. The optics allowed the pixel size adjustment ranging from 1 µm to 6.5 µm. For *T. molitor* specimens, the optical magnification was set to 2.4 resulting in an equivalent pixel size of 2.7 µm × 2.7 µm and a field-of-view of 5.3 mm × 5.3 mm. For *T. castaneum*, optical magnification was set to 4.3, resulting in an equivalent pixel size of 1.5 µm × 1.5 µm and a field-of-view of 3.0 mm × 3.0 mm. The sample was located at a distance of 23 m from the source, while the sample-to-detector distance was 150 mm and 100 mm for *T. molitor* and *T. castaneum* specimens, respectively. The projections were acquired using the free-space propagation imaging technique (Brombal et al. 2018), which allowed us to exploit phase-contrast effects leading to enhance contrast (edge-enhancement) at the boundaries between details with different compositions (Brombal 2020), considering the spatial coherence of the synchrotron source and its geometry. To optimise the signal-to-noise ratio, the propagation distances were defined after fixing the pixel size (Donato et al. 2022). To compensate for beam hardening effects, a 1.0 mm silicon filter was used to limit the high-energy portion of the X-ray beam, resulting in a spectrum with an average energy of about 20 keV. Because specimens of both species were longer than the vertical field-of-view, tomographic acquisitions required multiple (2 for *T. castaneum* and 4 for *T. molitor*) and partially overlapping scans. The exposure time for each projection was set to 0.250 s and 0.150 s for *T. molitor* and *T. castaneum,* respectively.

### Computer-based 3D-reconstruction

Image reconstruction allows the digital volumes of a single specimen to be created from the acquired projections. Image reconstruction was performed with a GPU-based filtered back-projection (FBP) algorithm using the SYRMEP Tomo Project (STP) software suite (Brun et al. 2015, 2017). A Shepp-Logan filter was used in the FBP reconstruction. Prior to image reconstruction, the projections were further processed by applying conventional flat-fielding, ring removal and phase-retrieval. The latter was based on the homogeneous transport of intensity equation (TIE-Hom) (Paganin et al. 2002). This algorithm works like a low-pass filter and allows to obtain images with a higher signal-to-noise ratio (compared to attenuation-based reconstruction) despite a loss of the edge-enhancement signal (Gureyev et al. 2017). The filter parameter δ/β was set to effectively regulate the amount of smoothing, as is common in experimental practice. For both species, this value was set to δ/β = 400, corresponding to a soft-tissue/adipose interface at the average energy of 20 keV, and calculated using the publicly available database (http://ts-imaging.science.unimelb.edu.au//Services//Simple//ICUtilXdata.aspx). The reconstructed volume yielded a 3D map that was essentially proportional to the linear attenuation coefficient of the sample (Brombal et al. 2018; Piai et al. 2019).

### Post-processing, image segmentation, and rendering

For each specimen, the full digital volume was obtained by combining (applying a linear blending) the individual stacks of reconstructed images. We used Avizo^®^ 3D for post-processing and segmentation. In brief, a region of interest containing the entire structure was selected and extracted for each system. Subsequently an “interactive threshold” module was applied to generate binary images that included as much of the identified structure as possible, while minimising any spurious ones. Then different morphological operators (such as “closing” and/or “opening” modules) were applied to refine the initial segmentation. The binary image was manually refined using Avizo's "Segmentation Editor", mainly with the "Lasso" and "Region growing" functions, and linear interpolation on volume was applied in the final step.

Avizo^®^ 3D was used for volume rendering of different beetle sections and animations were performed with the scientific visualisation software Drishti (Limaye 2012).

### Image analyses and measurements

Quantitative analysis for each organ of interest was carried out using the "Label Analysis'' module along with volume 3D measurement. Additionally, a length measurement for the alimentary canal was also performed through skeletonisation. For this purpose, the “Centerline Tree '' module was used, forcing the number of branches to 1 to obtain the longest segment connecting the endpoints of each structure. Finally, the segment obtained was smoothed using the "Smooth Line Set" module and then its length was measured using the "Spatial Graph Statistics'' module. Ganglion and alimentary canal volumetric measurements were estimated considering the segmentations shown in Fig. S3. Measurements are shown as mean ± standard error (SE).

## Results

The general external morphology of the male and female exarate pupae of *T. molitor* and *T. castaneum* is shown in Videos 1–4. The complete external structure of legs, mouthparts, and antennae appeared at the early stage and were not closely appressed to the body. The head was bent in a dorso-ventral forward position. Spiracles, located laterally in pleurites, opened to the tracheal vestibule in the body. Two spiracles were present on the mesothorax, two on the metathorax, while eight pairs were located on the first to eighth pleurite of the abdominal segments, respectively.

The internal morphology and its modifications over time are described below, separately for female and male of both species.

### Anatomy of *T. molitor* pupa

**Fig. 1:**
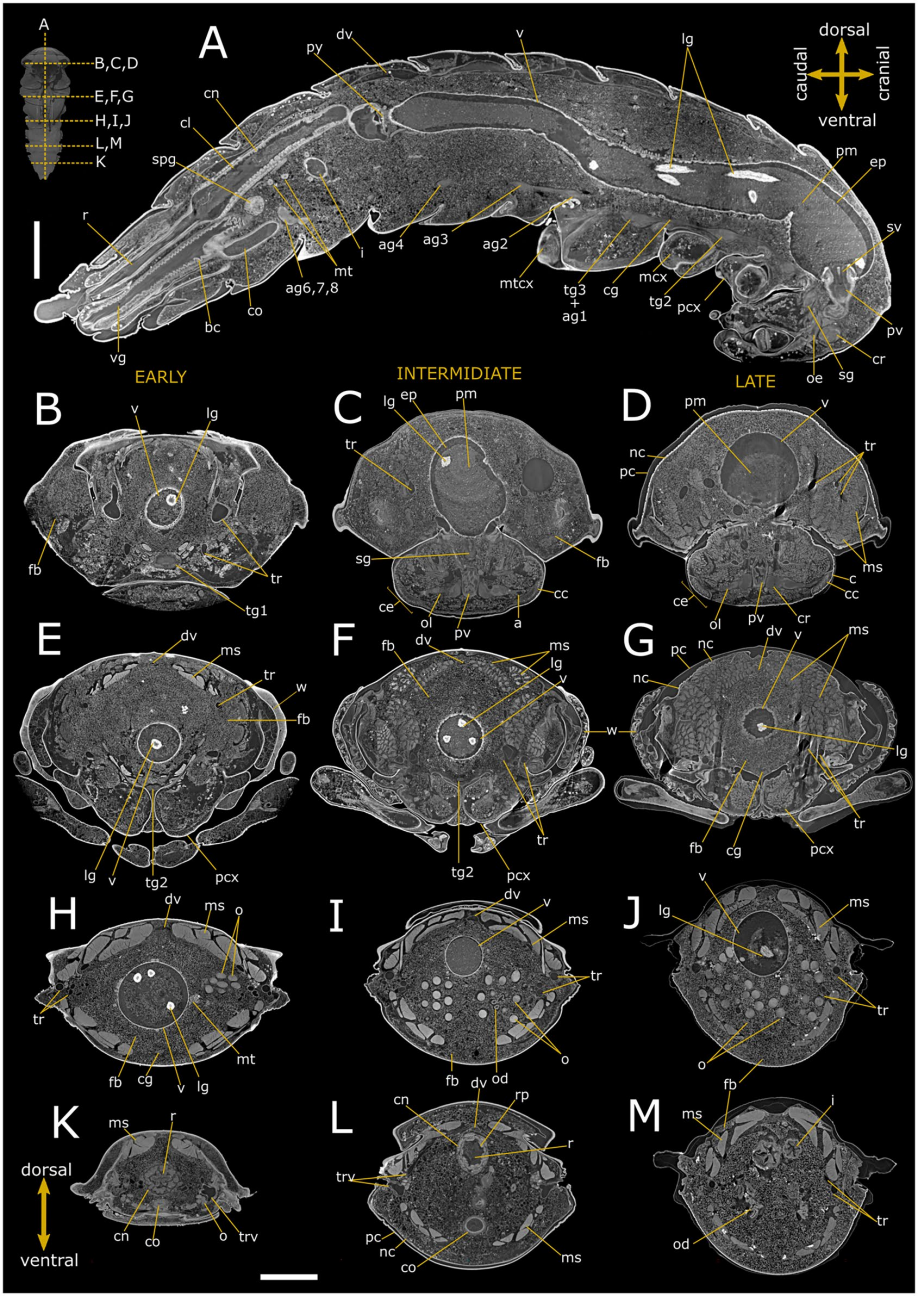
Synchrotron X-ray phase-contrast micro-CT images of *Tenebrio molitor* pupal stage. Two-dimensional longitudinal (**A**; 5-day-old) and virtual cross-sections of early (**B**, **E**, **H**, **K**; 1-day-old), intermediate (**C**, **F**, **I**, **L**; 5-day-old), and late (**D**, **G**, **J**, **M**; 8-day-old) pupal stage of female. The levels of the sagittal and cross sections are displayed in the top-left insert. a: axons; ag 2–4: abdominal ganglia 2,3 and 4; ag6, 7, 8: terminal abdominal ganglia fused to form a large caudal ganglion; bc: bursa copulatrix; c: cornea; cc: crystalline cones; ce: compound eye; cg: connective of ganglia; cl: colon; cn: cryptonephridial system; co: common oviduct; cr: cerebrum; dv: dorsal vessel; ep: ectoperitrophic space; fb: fat bodies; i: ileum; lg: larval gut; mcx: mesocoxa; ms: muscle; mt: malpighian tubules; mtcx: metacoxa; nc: newly formed cuticle; o: ovarioles; od:oviduct oe: oesophagus; ol: optic lobe; pc: pupal cuticle; pcx: procoxa; pm: peritrophic matrix; py: pyloric valve; pv: proventriculus; r: rectum; sg: suboesophagean ganglion; spg: spermathecal gland; sv: stomodeal valve; tg1 and 2: thoracic ganglia 1 and 2; tg3 + ag1: complex of thoracic ganglion 3 and abdominal ganglion 1; tr: tracheae; trv: tracheal vestibule; v: ventriculus; vg: vagina; w: wing. Scale bars:1 mm (**A**–**M**)

**Fig. 2:**
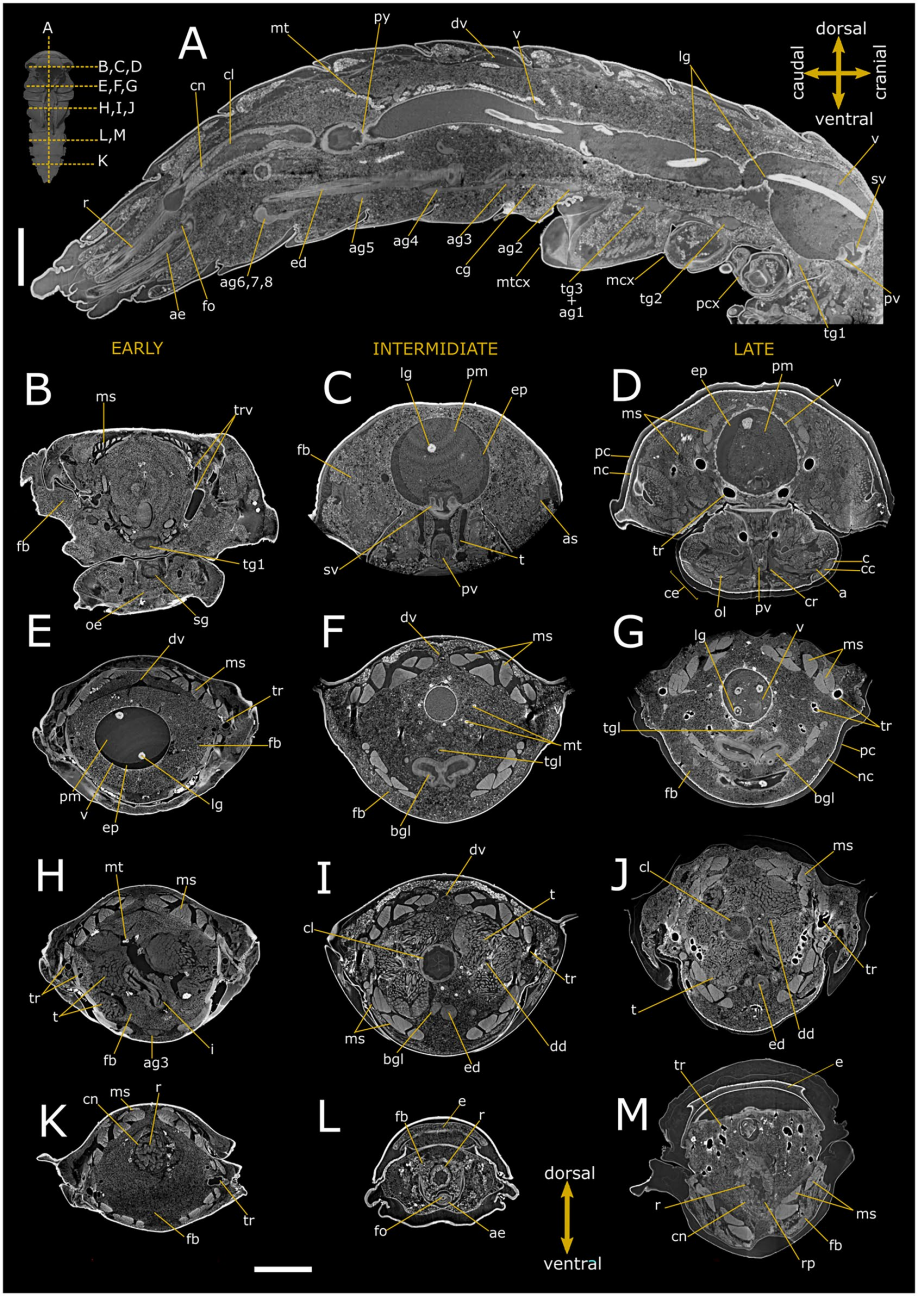
Synchrotron X-ray phase-contrast micro-CT images of *Tenebrio molitor* pupal stage. Two-dimensional longitudinal (**A**; 5-day-old) and virtual cross-sections of male pupae at early (**B**, **E**, **H**, **K**; 1-day-old), intermediate (**A**, **C**, **F**, **I**, **L**; 5-day-old), and late (**D**, **G**, **J**, **M**; 8-day-old) stage. The levels of the sagittal and cross sections are displayed in the top-left insert. a: axons; ae: aedeagus; ag 2–8: abdominal ganglia 2–8; as: atrium of spiracle; bgl: bean-shaped accessory glands; c: cornea of compound eye; cc: crystalline cones; cg: connective of ganglia; cl: colon; cn: cryptonephridial system; cr: cerebrum; dd: deferent duct; dv: dorsal vessel; ed: ejaculatory duct; ep: ectoperitrophic space; fb: fat bodies; fo: foramen; i: ileum; lg: larval gut; ms: muscle; mt: malpighian tubules; mcx: mesocoxa; mtcx: metacoxa; nc: newly formed cuticle; oe: oesophagus; ol: optic lobe; pc: pupal cuticle; pcx: procoxa; py: pyloric valve; pm: peritrophic matrix; pv: proventriculus; r: rectum; rp: rectal pads; sg: subesophagean ganglion; sv: stomodeal valve; t: testis; tgl: tubular accessory glands; tg1 and 2: thoracic ganglia 1 and 2; tg3 + ag1: complex of thoracic ganglion 3 and abdominal ganglion 1; tr: trachea; trv: tracheal vestibule; v: ventriculus. Scale bars:1 mm (**A**–**M**)

**Fig. 3:**
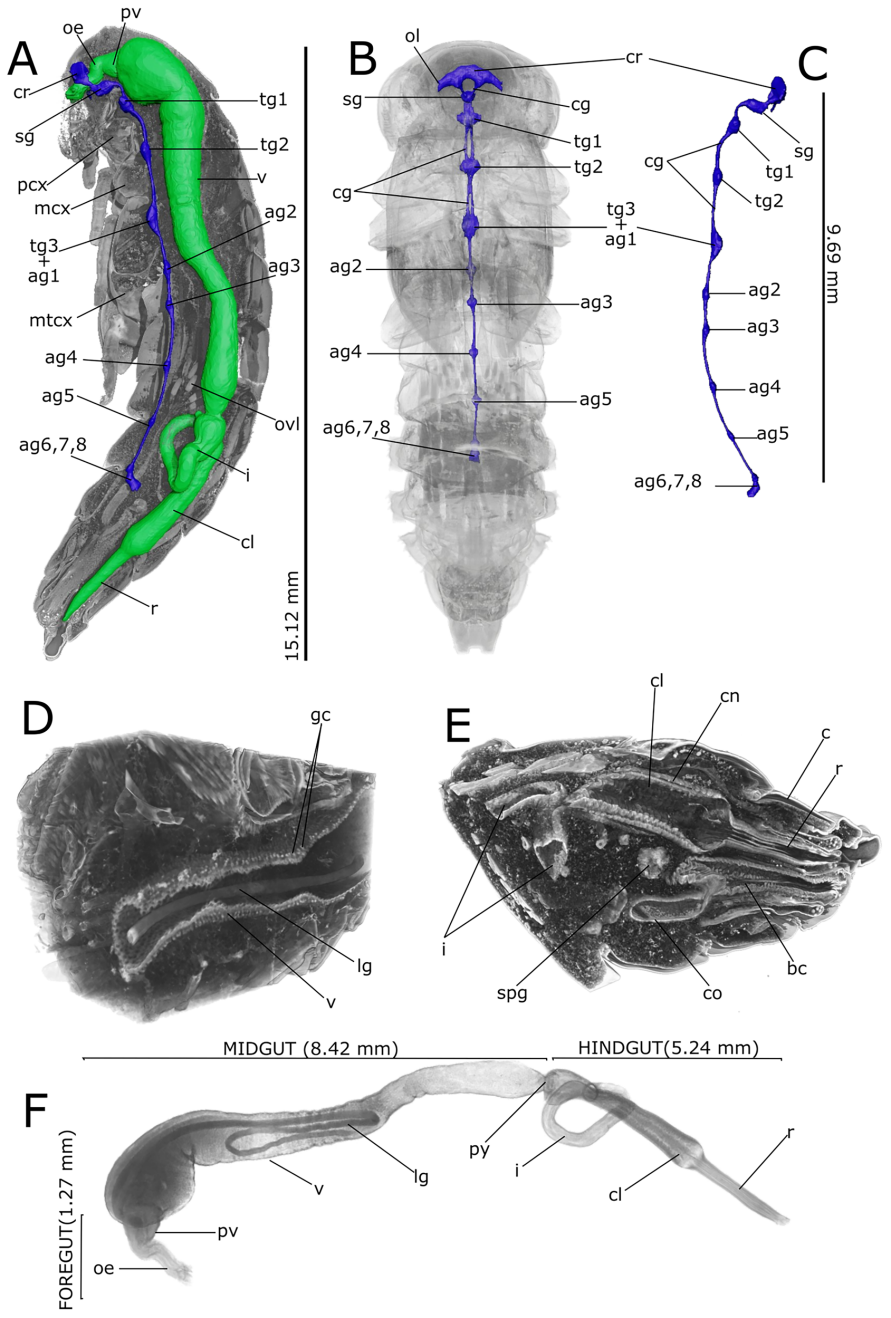
3D rendering and reconstructions of the nervous (**A**–**C**) and alimentary (**A**, **D**–**F**) systems of pupal stage in *Tenebrio molitor*. **A** Longitudinal rendering of the body showing the in situ reconstruction of the nervous system (blue) and alimentary canal (green) in a 5-day-old pupa. **B** Localization of the nervous system in the transparent rendered body, ventral view. **C** Lateral view of the segmented nervous system. **D** Detailed rendering images of the abdomen showing the larval gut in the lumen of pupal midgut. **E** Detailed rendering images of the abdomen showing the hindgut and reproductive apparatus of the female. **F** Lateral view of the segmented alimentary system. ag2-5: abdominal ganglia 2–5; ag6, 7, 8: terminal abdominal ganglia fused to form a large caudal ganglion; bc: bursa copulatrix; c: cuticle; cg: connective of ganglia; cl: colon; cn: cryptonephridial system; co: commune oviduct; cr: cerebrum; i: ileum; gc: gastric caecum; lg: larval gut; mcx: mesocoxae; mcx: mesocoxa; mtcx: metacoxa; oe: oesophagus; ol: optic lobe; py: pyloric valve; pv: proventriculus; r: rectum; sg: subesophagean ganglion; spg: spermathecal gland; tg1and 2: thoracic ganglia 1 and 2; tg3 + ag1: complex of thoracic ganglion 3 and abdominal ganglion 1; v: ventriculus

**Fig. 4:**
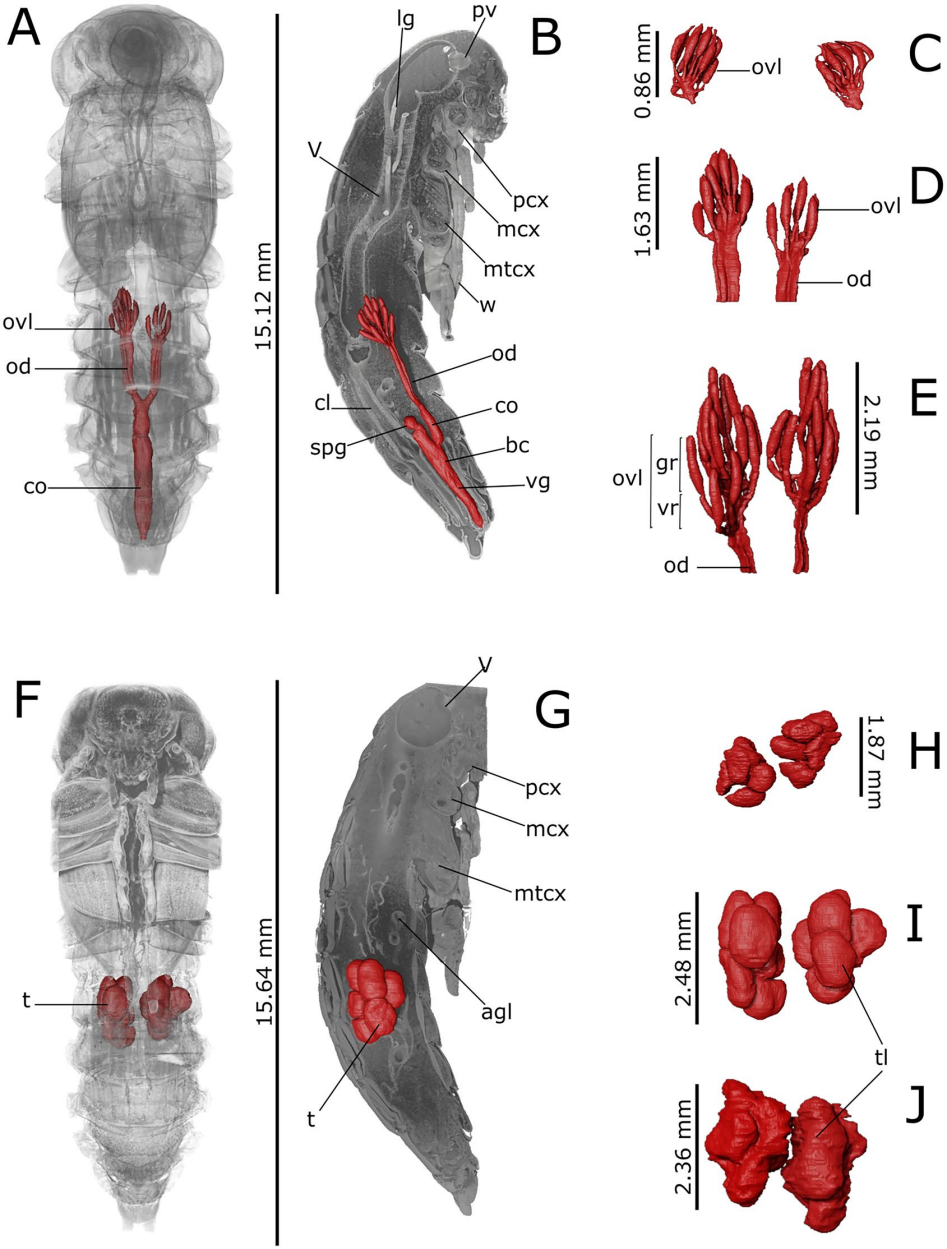
3D rendering and reconstructions of the reproductive system of *Tenebrio molitor* pupa. Reconstruction of the female’s reproductive system is shown in the ventral (**A**; 5-day-old pupa) and lateral (**B**; 5-day-old pupa) view. Volumetric reconstruction of the male’s testis is shown in the ventral (**F**; 5-day-old pupa) and lateral (**G**; 5-day-old pupa) view. Volumetric segmentation of ovariole (ovl) and testis (t) is shown in early (**C**, **H**; 1-day-old), intermediate (**D**, **I**; 5-day-old) and late (**E**, **J**; 8-day-old) stages. agl: accessory glands; bc: bursa copulatrix; cl:colon; co: common oviduct; gr: germinarium; lb: testis lobes; lg: larval gut; mcx: mesocoxae; mtcx: metacoxae; od: oviduct;; pcx: procoxae; t: testis; spg: spermathecal gland; pv:proventriculus; v: ventriculus; vg: vagina; vr: vitellarium; w: wings

Virtual dissections (Figs. 1 and 2), three-dimensional (3D) reconstructions, and renderings (Figs. 3 and 4) allow to describe in situ the anatomy of the nervous, digestive, and reproductive system and to estimate their volumetric sizes (Table 1) in both female and male pupae of *T. molitor* at different stages. Virtual sectioning revealed that the three main regions of the brain, i.e., protocerebrum, deutocerebrum, and tritocerebrum, were completed since the newly moulted pupa (Figs. 1A–C and 2A–C), while the nerves innervating the eyes, antennae, and mouth appendages became complete in 8-day-old pupae (Figs. 1D and 2D). A series of segmental ganglia lied ventrally to the body wall in a median position (Figs. 1A–C, F, H, 2A, B, D, G, H and 3A–C). There was one ventral suboesophageal ganglion, 3 thoracic, and 5 abdominal ganglia connected to each other by a pair of interganglionic connectives. The ganglia occupied less than 0.2 per cent of the body volume, and the cerebrum accounted for almost 40% of the nervous system volume (Table 1). The components of the visual system began to differentiate in the middle pupal stage (Fig. 1C); however, ommatidia and the connection to the optic lobes were clearly recognisable only at the late pupal stage (8 days; Figs. 1D and 2D).

**Table 1:** Volumetric measurements of segmented organs in *T. molitor* male and female (early, intermediate, late: 1, 5 and 8-day-old pupa respectively)

**Segmented structure**			**Volume** (mm^3^)			
**Alimentary canal**^a,b^	Foregut		2.08 ± 1.07			
	Midgut		4.26 ± 1.56			
	Hindgut		1.32 ± 0.13			
	Total		7.68 ± 0.46 (body: 146.25 ± 0.01)			5.25%
**Nervous system**^b^ (*intermediate*)	Cerebrum		9.70 × 10^–2^			
	Sub-oesophageal ganglion		2.76 × 10^–2^			
	Thoracic ganglia	1	3.45 × 10^–2^			
		2	2.89 × 10^–2^			
		3 + ag1	4.01 × 10^–2^			
	Abdominal ganglia	2	8.85 × 10^–3^			
		3	8.10 × 10^–3^			
		4	5.76 × 10^–3^			
		5	5.50 × 10^–3^			
		6, 7, 8	5.38 × 10^–3^ (+ ag5)			
	Total		2.45 × 10^–1^ (body: 141.55)	0.17%		
			**Female (ovary)**		**Male (testis)**	
**Gonads**^c^	early		(9.41 ± 0.75) × 10^–2^ (body: 147.91)	0.06%	(1.09 ± 0.01) (body: 121.54)	0.90%
	intermediate		(15.13 ± 2.46) × 10^–2^ (body: 141.55)	0.11%	(1.95 ± 0.02) (body: 149.29)	1.31%
	late		27.3 ± 0.07) × 10^–2^ (body: 138.90)	0.20%	(2.02 ± 0.06) (body: 176.71)	1.14%

^a^Alimentary canal measurements were assessed in both males and females at different stages (N = 3)

^b^The volumes of the alimentary canal and ganglia were estimated from the segmentation shown in Fig. S3

^c^Gonads are reported as the average between the right and left units (testis, ovary)

Sagittal (Figs. 1A and 2A) and transversal (Figs. 1B–M and 2B–M) stack of slices and reconstruction (Fig. 3D–F) showed the alimentary canal divided in three main regions: foregut, midgut, and hindgut, filling approximately 5.25% of the body volume. The foregut, formed by pharynx, oesophagus, and crop (Figs. 1A, C, D, 2B–D and 3A, F), appeared tubular in 1-day-old pupae. The proventriculus formed a valve at the beginning of the midgut and had a circular musculature that progressively thickened and became more defined from 1-day-old to 8-day-old pupae, at which time the stomodeal valve was clearly visible in virtual sectioning (Figs. 1A and 2A, C). The midgut was 4.26 ± 1.56 mm^3^ in volume and approximately 8.42 mm in length (Table 1; Fig. 3A, F), representing more than 55% of the volume of the entire intestine. The peritrophic matrix was visible leaning against the gut epithelium and contained the larval intestine, which was removed at the later stages. It appeared clearly visible in the lumen of the new alimentary canal, due to its strong attenuation contrast (Figs. 1A–H, J and 2A–E, G). In 8-day-old pupae, crypts appeared evident in the midgut wall forming gastric coeca (Figs. 1G, J, 2D, G and 3D). The hindgut, approximately 5.24 mm long, ran back to the distal part of the midgut (Table 1; Figs. 1A, K–M, 2A, H–M and 3A, F) and occupied about 17% of the gut volume. Virtual sectioning showed the pylorus, forming a pyloric valve (Figs. 1A, 2A and 3F), ileum, colon, and rectum (Figs. 1A, K–M, 2A, H–M and 3E, F). Malpighian tubules arose from the midgut-hindgut junction and projected into the haemocoelic cavity (Fig. S1E). Their distal part looped backwards into the cryptonephridial system at the anterior end of the rectum (Figs. 1A, 2A and 3E). The ileum was folded several times (Figs. 1A, 2A and 3A, E, F). 2D virtual sections (Figs. 1A and 3A) allowed to discriminate six rectal pads located on the inner surface of the rectum distal part (Figs. 1K–M and 2I–L).

Ovaries, each composed of eight ovarioles, and oviducts were evident in the abdomen laterally to the gut (Figs. 1H–J, 3A and 4A, B). They were incomplete in 1-day-old female pupae and the wall of median oviduct (Figs. 1H and 4C), vagina, and spermatheca were barely visible (Fig. 1K). These structures became complete from the fifth day of pupal stage (Figs. 1A, I and 4A, B, D). In 8-day-old pupae, the ovarioles were enveloped in a tunic and their distal filaments, germarium, and vitellarium were evident (Figs. 1J, 4E and S1A). The ovaries increase in size over time, threefold in volume from early to late pupal stage (Table 1).

The male reproductive system consisted of a pair of six lobed testes connected to the ejaculatory duct by the vas deferens in the early and intermediate pupal stage (Figs. 2H, F–J and 4F–J). The ejaculatory duct was connected to the aedeagus passing through the median foramen (Figs. 2A). The lobes of testis were well enveloped in a distinct epithelium and increased in volume by filling the space at the sides of the intestine in a dorso-ventral direction from the fifth day of the pupal phase (Figs. 2A, F–J, 4F–J and S1B), reaching about 1.23% of total volume between days 5 and 8 of pupation. They began to develop from the fifth day of the pupal phase (Fig. 4F, I) and increased in volume until the beginning of pupal-adult moulting (Fig. 4G, J). Accessory glands, one pair long, coiled, and tubular and one pair short and bean-shaped, opened into the ejaculatory duct at the major junction with the vas deferens glands (Figs. 2A, F–J and S1B).

### Anatomy of *T. castaneum* pupa

**Fig. 5:**
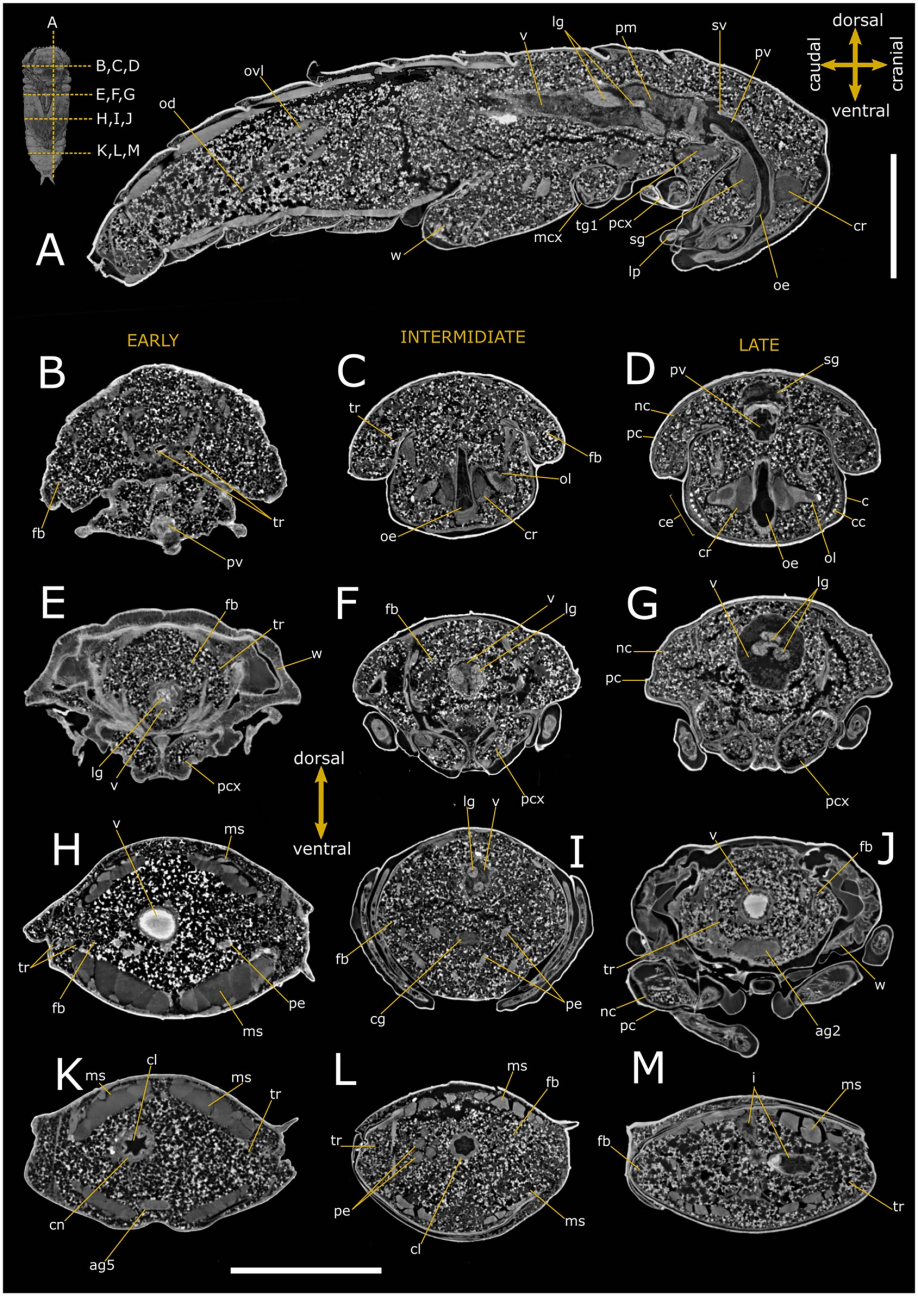
Synchrotron X-ray phase-contrast micro-CT images of *Tribolium castaneum* pupal stage. Two-dimensional longitudinal (**A**; 3-day-old) and cross-sections at early (**B**, **E**, **H**, **K**; 1-day-old), intermediate (**C**, **F**, **I**, **L**; 3-day-old), and late (**D**, **G**, **J**, **M**; 5-day-old) pupal stage of female. The levels of the sagittal and cross sections are displayed in the top-left insert. ag 2: abdominal ganglia 2; c: cornea; cc: crystalline cones; ce: compound eye; cg: connective of ganglion; cl: colon; cn: cryptonephridial system; cr: cerebrum; fb: fat bodies; i: ileum; lg: larval gut; lp: labial palp; mcx: mesocoxae; ms: muscle; nc: newly formed cuticle; od: oviduct; oe: oesophagus; ol: optic lobe; ovl: ovariole; pc: pupal cuticle; pcx: procoxae; pm: peritrophic matrix; pe: previtellogenic eggs; pv: proventriculus; sg: suboesophagean ganglion; sv: stomodeal valve; tg1 and 2: thoracic ganglia 1 and 2; tr: tracheae; v: ventriculus; w: wing. Scale bars: 500 μm (**A**–**M**)

**Fig. 6:**
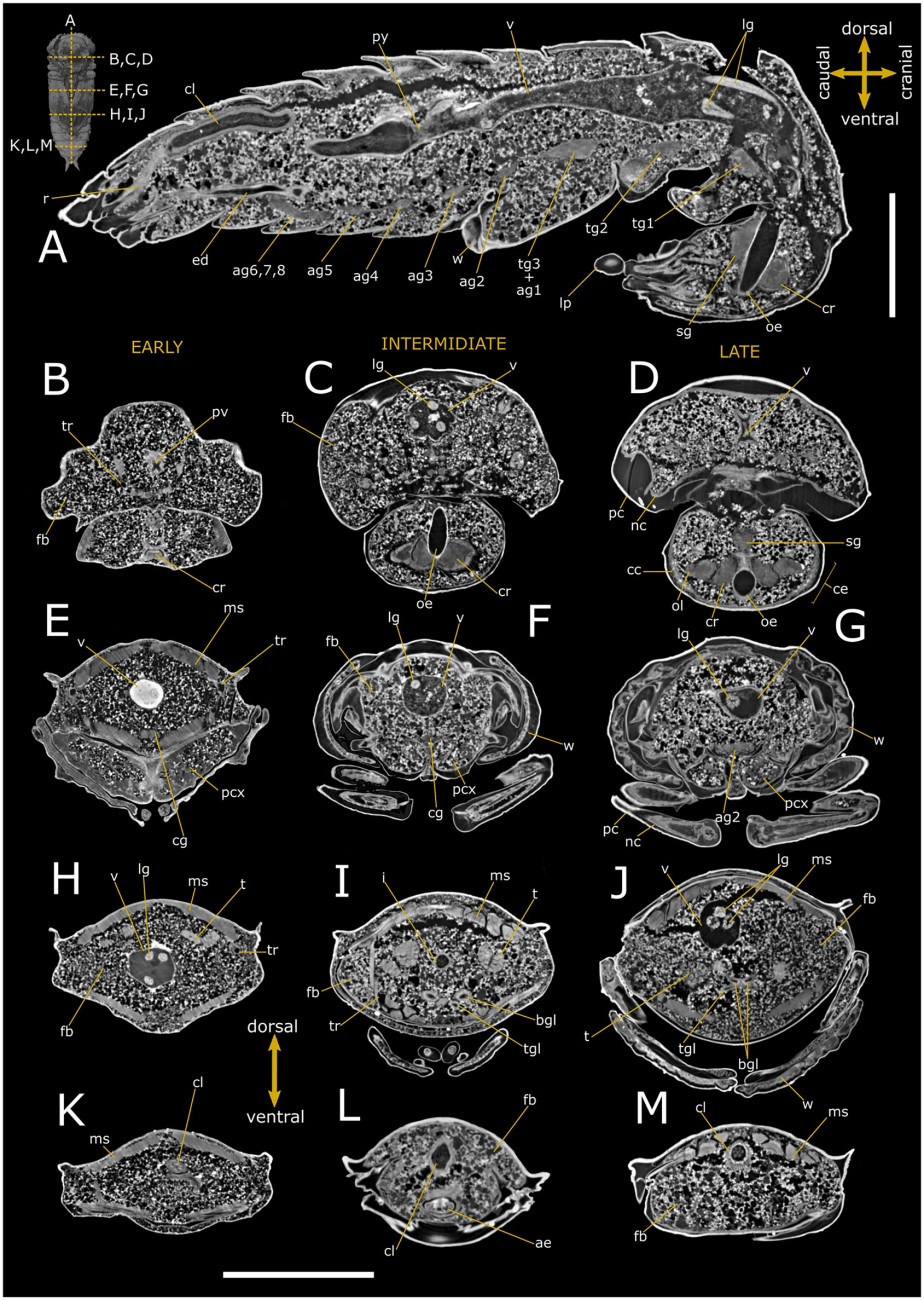
Synchrotron X-ray phase-contrast micro-CT images of *Tribolium castaneum* pupal stage. Two-dimensional longitudinal (**A**; 3-day-old) and cross-sections of male pupa at early (**B**, **E**, **H**, **K**; 1-day-old), intermediate (**C**, **F**, **I**, **L**; 3-day-old), and late (**D**, **G**, **J**, **M**; 5-day-old) stage. The levels of the sagittal and cross-sections are displayed in the top-left insert. ae: aedeagus; ag 2–5: abdominal ganglia 2–8; ag 6,7,8: complex of 6th, 7th, and 8th ganglia fused in a caudal ganglion; bgl: bean-shaped accessory glands; cc: crystalline cones; cg: connective of ganglia; cl: colon; cr: cerebrum; ed: ejaculatory duct; fb: fat bodies; i: ileum; lg: larval gut; lp: labial palp; ms: muscle; nc: newly formed cuticle; oe: oesophagus; ol: optic lobe; pc: pupal cuticle; pcx: procoxa; py: pyloric valve; pv: proventriculum; r: rectum; sg: subesophagean ganglion; t: testis; tg1-2: thoracic ganglia 1 and 2; tgl: tubular accessory gland; tr: trachea; v: ventriculus; w: wing. scale bars: 500 μm (**A**–**M**)

**Fig. 7:**
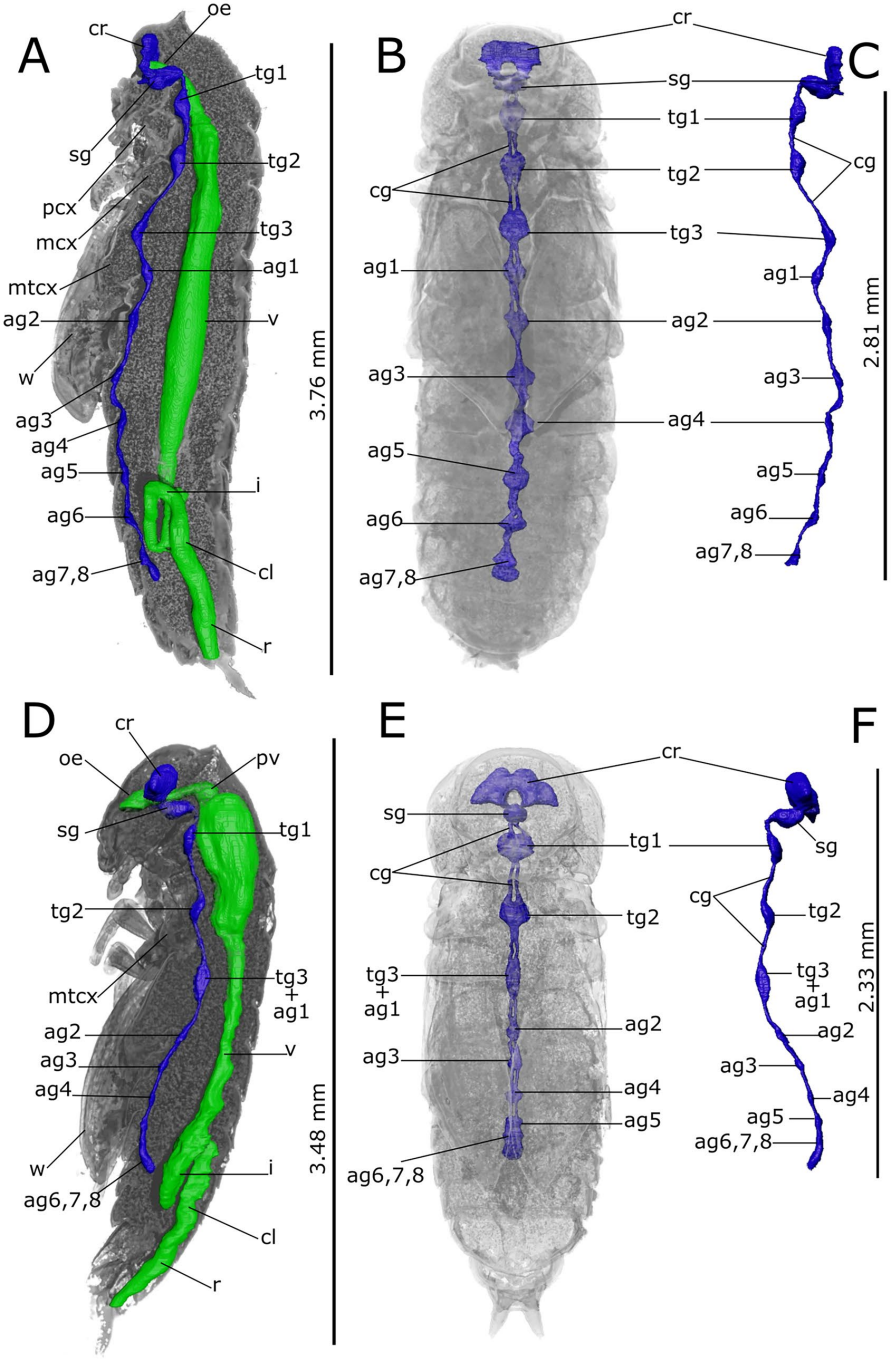
3D rendering and reconstructions of the nervous (blue) and alimentary (green) systems of *Tribolium castaneum* at early (**A**–**C**; 1-day-old), and late (**D**–**F**; 5-day-old) pupal stage. Lateral (**A**, **D**) and ventral (**B**, **E**) view of the nervous system volumetric segmentation. Lateral view of the segmented nervous system in early (**C**) and late (**F**) pupae. The reconstruction of the alimentary canal is shown in situ at early (**A**) and late (**D**) stage. ag2-8: abdominal ganglia 2–8; ag6, 7, 8: terminal abdominal ganglia fused to form a large caudal ganglion; cg: connective of ganglia; cl: colon; cr: cerebrum; i: ileum; oe: oesophagus; mcx: mesocoxa; mtcx: metacoxa; pcx: procoxa; pv: proventriculus; r: rectum; sg: subesophagean ganglion; tg1-3: thoracic ganglia 1–3; tg3 + ag1: complex of thoracic ganglion 3 and abdominal ganglion 1; v: ventriculus; w: wing

The central body of protocerebrum, including corpus pedunculatum and central complex, became evident in the 1-day-old pupa of both females and males (Figs. 5B, 6B and 7A–C; Table 2), while lobes of deutocerebrum and tritocerebrum appeared well defined in virtual sectioning from day 3 of the pupal stage (Figs. 5A, C and 6A, C), with a more than threefold increase in volume (Table 2). The optic lobes, that represented the lateral extensions of the protocerebrum to the compound eyes, and ommatidia became complete in 5-day-old pupa (Figs. 5D, 6D and 7D–F). Segmented ganglia were evident in 3D reconstruction and virtual sagittal slices, lying ventrally the body wall in a median position (Figs. 5A, D, I–K, 6A, D, E, G and 7A–F). They were twelve in 1-day-old pupae, arranged in one ventral suboesophageal ganglion connected to brain by circumesophageal connectives, 3 thoracic, and 8 abdominal ganglia connected each other by a pair of interganglionic connectives (Figs. 7A–C and S3C). In 3-day-old pupae, the metathoracic ganglion and the first abdominal ganglion began to appear fused as well as the 6th, 7th, and 8th abdominal ganglia, which merged completely in the 5-day-old pupa (Fig. 7D–F), filling 0.56% of the body volume in the 1-day-old pupa and 0.76% in the last stage (5-day-old pupa; Table 2). The cerebrum accounted for approximately 51% of the total volume of the nervous system at this stage.

**Table 2:** Volumetric measurements segmented organs in *T. castaneum* male and female (early, intermediate, late: 1, 3 and 5-day-old pupa respectively)

**Segmented structure**			**Volume** (mm^3^)			
**Alimentary canal**^a,b^	Foregut		(0.26 ± 0.13) × 10^–2^			
	Midgut		(4.81 ± 0.77) × 10^–2^			
	Hindgut		(1.52 ± 0.14) × 10^–2^			
	Total		(6.62 ± 1.01) × 10^–2^ (body: 1.69 ± 0.09)		3.92%	
**Nervous system**^b^			*Early*		*Late*	
	Cerebrum		1.94 × 10^–3^		6.80 × 10^–3^	
	Sub-oesophageal ganglion		1.52 × 10^–3^		1.36 × 10^–3^	
	Thoracic ganglia	1	1.0 × 10^–3^		1.30 × 10^–3^	
		2	0.80 × 10^–3^		1.01 × 10^–3^	
		3 + ag1	0.84 × 10^–3^ (tg3); 0.42 × 10^–3^ (ag1)		1.02 × 10^–3^	
	Abdominal ganglia	2	0.36 × 10^–3^		0.21 × 10^–3^	
		3	0.35 × 10^–3^		0.19 × 10^–3^	
		4	0.38 × 10^–3^		0.19 × 10^–3^	
		5	0.35 × 10^–3^		0.16 × 10^–3^	
		6, 7, 8	0.26 × 10^–3^ (ag6); 0.67 × 10^–3^(ag7,8)		1.02 × 10^–3^	
	Total		8.89 × 10^–3^ (body: 1.60)	0.56%	13.27 × 10^–3^ (body: 1.78)	0.76%
			**Female (ovary)**		**Male (testis)**	
**Gonads**^c^	*Early*		nd		nd	
	*Intermediate*		(1.30 ± 0.04) × 10^–3^ (body: 1.60)	0.08%	(1.93 ± 0.03) (body: 1.71)	0.11%
	*Late*		(1.88 ± 0.11) × 10^–3^ (body: 1.78)	0.11%	(1.05 ± 0.02) (body: 1.39)	0.08%

*nd* not determined

^a^Alimentary canal measurements were assessed in both males and females at different stages (N = 2)

^b^The volumes of the alimentary canal and ganglia were estimated from the segmentation shown in Fig. S3

^c^Gonads are reported as the average between the right and left units (testis, ovary)

The alimentary canal consisted of three main regions, namely the foregut, midgut, and hindgut. Foregut and midgut of 1-day-old pupae appeared as a single tubular-shaped structure separated by the stomodeal valve and the intestine of larval stages was featured by higher attenuation and evident in the lumen (Figs. 5B, E, H, K, 6B, E, H, K and 7A). Pharynx, oesophagus, and crop and proventriculus were structurally distinguished in 5-day-old pupae (Figs. 5D, 6D and 7D), they filled (0.26 ± 0.13) × 10^–2^ mm^3^ in volume and measured about 0.59 mm in length. The stomodeal valve partitioned the proventriculus from the beginning of the midgut and was well defined in 5-day-old pupae (Figs. 5D, 6D and 7D), advanced pupal stage. The midgut represented 4.81 ± 0.77 × 10^–2^ mm^3^ in volume and was about 1.86 mm long. It was the largest part of the gut, reaching approximately 73% of the alimentary canal (Table 2) and was completely renewed and formed a layer surrounding the larval alimentary canal (Figs. 5A, F–J and 6E–H, J). In 5-day-old pupae, crypts appeared evident in the midgut wall forming gastric coeca. Virtual sectioning showed the hindgut approximately 1.04 mm long consisting in ileum, colon and rectum (Figs. 5K–M and 6A, K–M), separated from the distal part of the midgut by a pyloric valve. This part of the gut appeared complete already in the 1-day-old pupa (Figs. 5K and 7A). The distal part of Malpighian tubules looped backwards into the cryptonephridial system and was well defined in the anterior end of the rectum wall. The ileum was folded several times and continued into the colon with a thicker muscular wall (Fig. 5K and 7A). Virtual sections discriminated six rectal pads in the rectum structure (Fig. 7D) located on the inner surface at the distal part of the rectum (Figs. 5K–M and 6K–M).

**Fig. 8:**
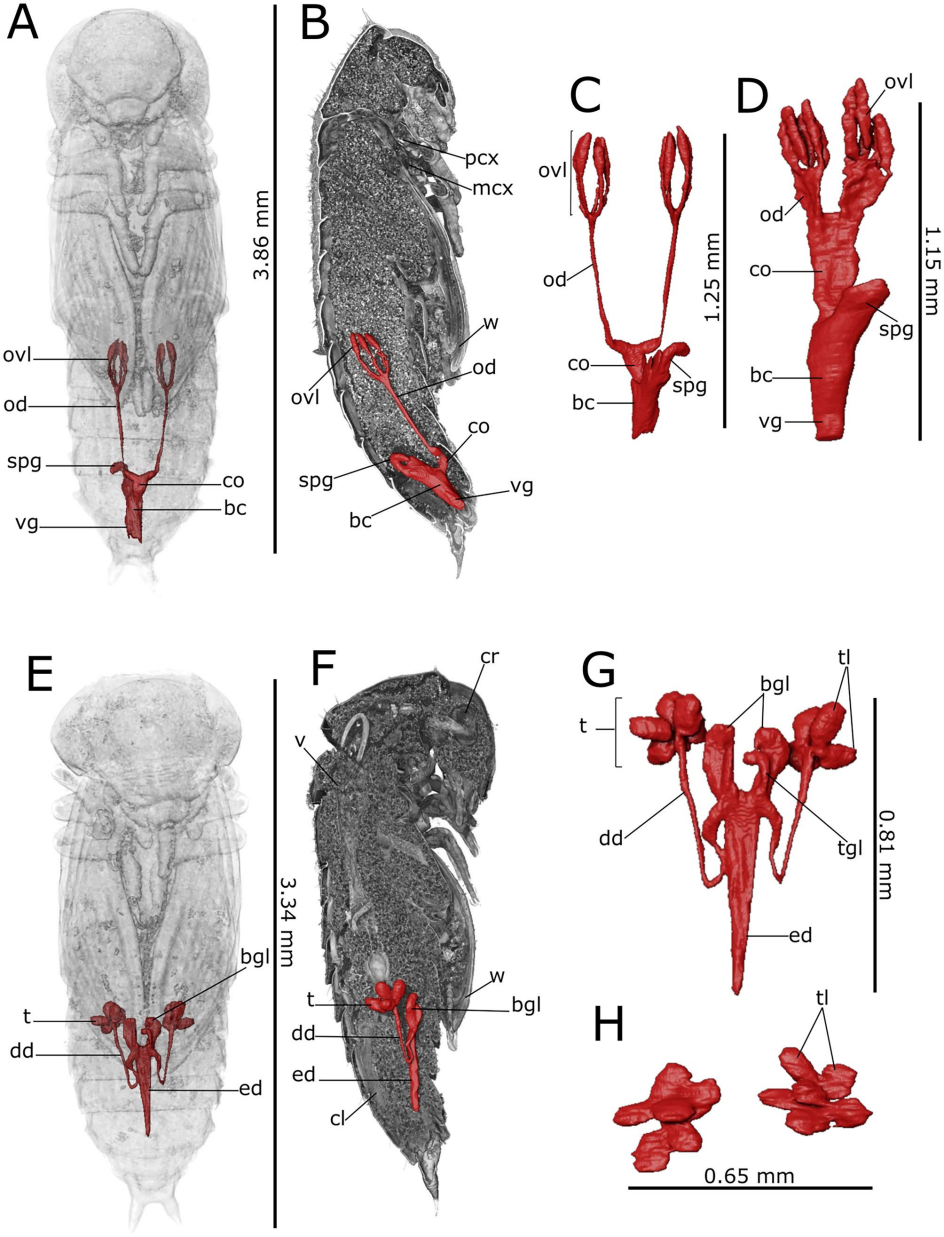
3D rendering and reconstructions of the *Tribolium castaneum* reproductive system. Reconstruction of the female’s reproductive system is shown in the ventral (**A**; 3-day-old pupa) and lateral view (**B**; 3-day-old pupa). Ventral (**E**; 3-day-old pupa) and lateral view (**F**; 3-day-old pupa) of male’s testis. Volumetric segmentation of ovaries (ovl) and testis (t) is shown in pupae at the intermediate (**C**, **G**; 3-day-old) and late (**D**, **H**; 5-day-old) stage. bc: bursa copulatrix; bgl: bean-shaped accessory gland; co: common oviduct; dd: deferent duct; ed: ejaculatory duct; mcx: mesocoxa; od: oviduct; ovl: ovariole; pcx: procoxa; spg: spermathecal gland; t: testis; tgl: tubular accessory gland; tl: testis lobules; vg: vagina; w: wing

Each ovary was composed of six telotrophic ovarioles and connected to the median oviduct by the lateral oviduct in 5-day-old pupae. Virtual sagittal sections showed that these structures were incomplete in one-day-old female pupae and the wall of median oviduct, vagina, and spermatheca were absent (Fig. 5H, K). They became complete from the 3rd day of pupal stage (Figs. 5A and 8A–C), at an intermediate phase. In 5-day-old pupae, the later stage, the ovarioles were enveloped in their tunic, but their distal filaments, germarium, and vitellarium did not appear clearly identifiable yet (Fig. 8D). The increase in ovarian volume was about 27% comparing the intermediate to the late stage (Table 2).

Virtual dissections and 3D reconstructions showed a pair of six lobed testes in 1-day-old pupae connected to the ejaculatory duct by the vas deferens. The lobes of testis underwent a reorganisation and shape change over pupal stage, by filling the space at the sides of the intestine in a dorso-ventral direction from the third day of the pupal phase (Figs. 6A, I, J and 8E–G). Accessory glands were one pair long and tubular and one pair short and finger-shaped that opened into the ejaculatory duct at the major junction with the vas deferens glands (Figs. 6A, I, J and 8E–H). They appeared well-defined by day 3 of the pupal phase and increased in volume until the beginning of the pupal-adult moulting phase.

### Other structures

The diaphanous wing buds appeared flattened at the thoracic suture between the pleuron and the tergum, and elongated at the lateral side of the body (Figs. 1E–G, 5E and S2A, B). Flight muscles became evident in the virtual sections at the end of the pupal stage in both species (Figs. 5J and S1A, B).

New endocuticle formation began at the half of the pupal phase for both species, but in the virtual sections the space between the pupal cuticle and the newly formed cuticle became clearly evident in the last stage (Figs. 1D, G, J, M, 2D, J, M, 5D, G, J, M and S2A, B). The apolysis of the pupal cuticle, and the formation of epicuticle and exocuticle of the adulthood were completed at the end of the pupal stage.

The reconstruction of the fat body, tracheae, circulatory system, and muscles was not possible because the lack of sufficiently detailed differences in contrast. However, these structures were indicated in the virtual sections where they were evident.

## Discussion

D﻿ata reported in this study provide the first description of the internal anatomy of *T. molitor* and *T. castaneum* pupae based on high resolution images, obtained by using SR-PhC micro-CT. This technique allowed us to perform virtual dissections of the internal structures of these insects in transversal, sagittal, and frontal body planes, revealing in situ anatomical changes over time. The volume renderings provided adequate morphological information on the remodelling of the alimentary canal and nervous system, as well as reproductive system shaping in females and males of both species. Despite its adaptive value in holometabolous insects, little information regarding the inner anatomy of the pupal stage has been reported in the literature. The main reason for this fragmentation of knowledge may be methodological limitations associated with classical histology techniques such as cut compression, distortion, loss and discontinuity of sections, section loss and discontinuity, and staining artefacts. These aspects prevent the accurate extraction of information necessary for generating images of the ongoing construction process of soft organs and the spatial arrangement of the fat body in the pupal hemocoel. Micro-CT is an innovative, promising method for investigating the internal anatomy of insects without time-consuming histological protocols, including dissections and serial sectioning (Betz et al. 2007; Gutiérrez et al. 2018; Sombke et al. 2015). Accordingly, it has been applied for studying the morphology of different organs in adult insects, mainly focusing on the anatomy of the alimentary canal (Alba-Alejandre et al. 2018, 2019, 2020), as well as the nervous (Donato et al. 2021; Giglio et al. 2022; Smith et al. 2016) and reproductive (Alba-Alejandre et al. 2020; Küpper et al. 2019; Vommaro et al. 2022) systems. Recently, virtual sectioning and 3D volumetric reconstructions, obtained from X-ray tube source, have been successfully used to describe the internal structures of the pupal stage and their volumetric and structural variations over time in a limited number of species (Helm et al. 2018; Ikegami et al. 2023; Lowe et al. 2013; Nur et al. 2019; Zhao et al. 2020). From a technical perspective, a significant advantage of the SR-PhC micro-CT analysis applied in our study is represented by the lack of sample staining by highly toxic chemicals such as uranyl acetate, iodine, phosphotungstic acid, and osmium tetroxide (Betz et al. 2007; Metscher 2009; Smith et al. 2016), while maintaining quality sample absorption and image contrast (Donato et al. 2021; Giglio et al. 2022; Heethoff and Cloetens 2008; Vommaro et al. 2022, 2023a). The application of this technique for morphological studies of insects, including miniaturised species such as *T. castaneum*, allows the preservation of the overall spatial architecture of organs in their native orientation and the possibility to perform accurate, quantitative, morphometric measurements based on high-resolution image datasets and segmentation tools. Thus, further studies involving this powerful technique can reveal significant developmental differences among species, to understand evolutionary morphological and ecological adaptations in the temporal shaping of organs during the pupal stage of holometabolous insects.

To date, extensive attention has been paid to the anatomy of the insect nervous system, especially the brain that controls vital, behavioural, and functional activities (Rother et al. 2021; Smith et al. 2016). Although the general architecture of cerebrum is well conserved, it was found that variations in the volume and neuroanatomy of brain regions are correlated with the ecological and ethological requirements of each species (Carle 2021; Chittka and Niven 2009; Eriksson et al. 2019; Immonen et al. 2017). Indeed, a recent study comparing 63 beetle species belonging to 22 different families demonstrated the existence of a correlation between the species-specific organisation of the antennal lobe and lifestyle and feeding habits (Kollmann et al. 2016). Standardised information on the brain of *T. castaneum* adults have been obtained using 3D reconstructions based on confocal laser scanning microscopy (Dreyer et al. 2010; Farnworth et al. 2022; Hunnekuhl et al. 2020) and micro-tomography (Vommaro et al. 2023a) imaging techniques. However, the remodelling of the nervous system during postembryonic development has received less attention (Farnworth et al. 2022; Koniszewski et al. 2016; Wegerhoff and Breidbaeh 1992). Indeed, limited studies conducted on insects are available and concern the neurogenesis during the pupal stage of *Drosophila melanogaster* (Li and Hidalgo 2020) and *Apis mellifera* (Farris et al. 1999). Our analyses indicate that the nervous system shows distinct timing of differentiation and reorganisation during development, despite the taxonomic relationship of the studied species. SR-PhC micro-CT allowed us to reconstruct the brain during pupal development, highlighting its structural modification and volumetric increase, mainly of the optic lobes, that accompany the transition from larval stemmata to adult compound eyes. A drastic change takes place in *T. castaneum* during pupation, with an increase in the volume of the cerebrum and a reduction of the ganglia due to their fusion. Indeed, virtual sections revealed that, during metamorphosis of *T. castaneum*, the reorganisation of metathoracic ganglion occurs, incorporating the first abdominal ganglion, while sixth, seventh, and eighth abdominal ganglia are fused in a terminal ganglion. Conversely, in *T. molitor* these modifications of ganglial nervous system segmentation occur at the pre-pupal stages, as described by Breidbach (1987). In virtual dissections, the anatomical structure of the adult nervous system can be observed already at early pupal stages.

In Coleoptera, larval and adult digestive systems exhibit differences when both stages are characterised by a variation in the feeding habits (Chapman 2012; Crowson 1981; Engel and Moran 2013) involving a deep reorganisation of gut features during pupal stage. Unexpectedly, although the omnivorous diet of red flour and yellow mealworm larvae is highly comparable to that of adults, both species undergo morphological changes in the gut during pupal stage. The volume of the foregut increases, and the crop appears in its distal part. Furthermore, cellular proliferation in the midgut region with the appearance of regenerative crypts or diverticula on its basal surface results in the replacement of the larval midgut (Nardi and Bee 2012; Parthasarathy and Palli 2008). Thus, the crop and midgut regenerative cripts, absent in *T. castaneum* (Ameen and Rahman 1973) and *T. molitor* (Cristofoletti et al. 2001) larvae, are present in adult and facilitate an increased food storage capability and nutrient digestion and absorption, respectively (Caccia et al. 2019; Gigliolli et al. 2015; Holtof et al. 2019; Nardi and Bee 2012). This suggests that adults, which are more mobile than larvae, undergo a change in foraging strategy (Dawson 1977; Pointer et al. 2021). In contrast, the structure of the posterior intestine, including cryptonephridial system and rectal pads, does not appear to be significantly remodelled during the pupal phase, comparing larvae to adults (Grimstone et al. 1968; King and Denholm 2014; Koefoed 1971; Ramsay 1976; Vommaro et al. 2023a). This indicates that there is a common physiological adaptation in excretion and osmoregulation processes to minimise water loss and salt recovery, as both larvae and adults of these species have mainly access to dry food. Nevertheless, the perirectal tube is longer in *T. molitor* adults than in the larvae. Additionally, each rectal pad consists of five specialized epithelial cells showing a microvillar zone on the apical surface. In larvae, these cells are closely apposed while in adults they are spaced out in the perirectal area (Noble-Nesbitt 1990).

The reproductive system is a typical adult structure, absent during the larval stage of holometabolous insects that is fully developed at the pupal stage (Chapman 2012). The fragmentary information available in the literature about male and female reproductive organs in the red flour and mealworm beetles concerns baseline data for developing strategies to control fecundity and reproductivity of these pest species. For example, pupal irradiation could be employed to interrupt gonad maturation (Tungjitwitayakul et al. 2022). Previous immunohistochemical and ultrastructural analyses provided an extensive morphological description of the telotrophic meroistic ovariole in *T. castaneum* pupae (Büning 1979a, b; Takaki et al. 2020; Trauner and Büning 2007; Srivastava 1956) and *T. molitor* (Ullmann 1973). Moreover, the morphology of the testicular development (Menon 1969) and accessory glands (Happ and Happ 1982) has been documented for the pre-imaginal stages of mealworm. Recent studies have also concentrated on assessing gene expression related to the development of reproductive structures in the red flour beetle (Li et al. 2023; Shukla and Palli 2013). The description of the general anatomical modifications during post-embryonic development herein provided is in line with the information reported in the literature. The novelty herein presented is the use of SR-PhC micro-CT that provides a helpful support to describe in situ the sequence of events in both female and male reproductive organ development and obtain volumetric measurements. Virtual sections and 3D reconstruction set out the anatomical transformations of easily recognised structures such as testes and their accessory glands, as well as telotrophic ovarioles. This technique allows a comparative analysis between the volume of ovaries and testes in the pupae and adults of *T. castaneum*, reported in previous studies, revealing a substantial increase due to post-pupal maturation, with a gain of over 96% in volume. However, micro-CT is not the appropriate technique to acquire information about germ cells during preimaginal development due to the low resolution at the ultrastructural scale (Vommaro et al. 2023a).

Finally, our data allowed, for the first time, to visualise in situ the moulting process involved in the synthesis of new cuticle, accompanied by the shedding of pre-existing cuticle, which occurs at 50% of the pupal phase duration in both species. The cuticle of pupal stage is a multifunctional device that protects insects from dehydration and predators, and constitutes a physical barrier to prevent pathogen entry (Vincent and Wegst 2004). Moreover, it can be a secondary sexual character due to its colour and thickening (Prokkola et al. 2013; Rolff et al. 2005). However, there are only a few aspects of the pupal-adult moult phase that have been studied, such as the proteomic composition of the moulting fluid (Qu et al. 2014) or the hormonal control of the epidermal cells (Besson-Lavoignet and Delachambre 1981; Truman 2019). The pattern of pre- and post-ecdysis proteins during metamorphosis has been identified in mealworm beetles (Baernholdt and Andersen 1998; Bouhin et al. 1992; Delbecque et al. 1978; Roberts and Willis 1980), while genes involved in cuticle morphology have been described in the red flour beetles (Arakane et al. 2005). Thus, it would be of great interest to study morphological and molecular aspects of moulting during this transitional phase.

## Conclusions

Results herein reported represent the first characterization of the internal anatomical structure during the pupal stage of females and males in both *T. molitor* and *T. castaneum*. In general, there were no significant differences in gross morphological features between the species studied, as observed in virtual section stacks of the pupal stage at different ages. Differences were only observed in the timing of gut and nervous system remodelling and reproductive system shaping, which are likely related to the ecological adaptation of each species according to the duration of their life cycle (Fig. S4). Our findings support the usefulness of SR-PhC micro-CT as a valuable tool to investigate anatomical modifications during the pupal stage of holometabolous insects. The method significantly reduces time and number of samples required for dissections and sectioning, and for the acquisition of 2D and 3D images. Moreover, its non-destructive properties allow a high degree of repeatability and reliable quantitative analysis. However, this approach has some limitations such as the limited detection of tissue and cell-level changes throughout the pupal stages, suggesting that a combination of this technique with histological or electron microscopy analyses could lead to a complete overview of the data. Moreover, the use of synchrotron facilities remains a significant challenge for researchers conducting large dataset studies. Challenges include limited beamline availability, scheduling constraints, and the need of specialized expertise. However, compact setups of advanced X-ray sources and phase contrast methods in research laboratories can achieve resolutions comparable to synchrotron systems (Romell et al. 2021; Windfelder et al. 2023).

Despite mealworm and red flour beetles are well studied as models in experimental biology and because of the economic concern as pests of stored products, data concerning organ shaping and reorganization in the pupal stage are scarce, fragmentary, and incomplete. In this scenario, our study confirmed that descriptive morphology cannot be relegated to a secondary role in systematic and ecological studies, and it is essential for understanding the form and function of organisms in evolutionary and developmental biology, as noted in previous studies (Wanninger 2015; Wipfler et al. 2016). Videos and rendering of structures highlight the importance of imaging in morphological research to facilitate comparison between taxa and teaching entomology.

### Supplementary information

Below is the link to the electronic supplementary material.

Supplementary Fig. S1 Internal anatomy of *Tenebrio molitor* pupae. Two-dimensional PhC micro-CT-based longitudinal of female (A) and male (B) 8-day-old showing gonads, and cross sections at cranial (C; 8-day-old male), thoracic (D; 5-day-old female) and abdominal (E; 5-day-old female) level. bgl: bean-shaped accessory glands; an: antennomere; anv: antennal nerve; cg: connective of ganglia; cr: cerebrum; dd: deferent duct; dv: dorsal vessel; ep: ectoperitrophic space; f: apical filaments; fb: fat bodies; gr: germinarium; i: ileum; lg: larval gut; mcx: mesocoxa; ms: muscle; mt: malpighian tubules; mtcx: metacoxa; nc: new cuticle; od: oviduct; pc: pupal cuticle; pcx: procoxa; pe: pre-vitellogenic egg; pm: peritrophic matrix; pv: proventriculus; sv: stomodeal valve; t: testis; tr: tracheae; v: ventriculus; vr: vitellarium; wms: wing muscles. Scale bars:1 mm (A–E) (TIFF 15091 KB)

Supplementary Fig. S2 3D volumetric rendering of *Tenebrio molitor* (A; 5-day-old female) and *Tribolium castaneum* (B; 5-day-old male) pupal stage, showing the newly formed cuticle (nc) enwrapped by the pupal cuticle (pc). (C) Segmented alimentary systems of Tribolium castaneum at late pupal stage (5-day-old female). an: antennomere; ce: compound eye; cl: colon; i: ileum; lg: larval gut; ms: muscle; mxp: maxillary palp; oe: oesophagus; om: ommatidia; pv: proventriculus; py: pyloric valve; r: rectum; sv: stomodeal valve; v: ventriculus; w: wing (TIFF 1757 KB)

Supplementary Fig. S3 3D reconstruction of the segmented pupal alimentary (A, B) and nervous systems (C, D) of *Tenebrio molitor* (A and C; 5-day-old) and *Tribolium castaneum* (B and D; 5-day-old) to obtain volumetric measurements as reported in Table 1 and 2. ag2-5: abdominal ganglia from 2 to 5; ag6, 7, 8: terminal abdominal ganglia fused to form a large caudal ganglion; cl: colon; cr: cerebrum; i: ileum; oe: oesophagus; pv: proventriculus; r: rectum; sg: subesophagean ganglion; tg1 and 2: thoracic ganglia 1 and 2; tg3 + ag1: complex of thoracic ganglion 3 and abdominal ganglion 1; v: ventriculus (TIFF 723 KB)

Supplementary Fig. S4 Schematic timeline of the events occurring at the pupal stage of *T. molitor *(Tm) and *T. castaneum* (Tc). The chronology refers to the early (day 1), intermediate (day 3 Tc; day 5 Tm), and late (day 5 Tc; day 8 Tm) stage. The overlapping area shows common developmental stages of the two species related to general morphology, alimentary canal, nervous and reproductive systems, while non-common events are species-specific (TIFF 3125 KB)

Supplementary Video 1 3D reconstruction of *Tenebrio molitor* 5-day-old pupa female (intermediate pupal stage) (MP4 5415 KB)

Supplementary Video 2 3D reconstruction of *Tenebrio molitor* 5-day-old pupa male (intermediate pupal stage) (MP4 14978 KB)

Supplementary Video 3 3D reconstruction of *Tribolium castaneum* 1-day-old pupa female (early pupal stage) (MP4 6883 KB)

Supplementary Video 4 3D reconstruction of *Tribolium castaneum* 1-day-old pupa male (early pupal stage) (MP4 13165 KB)

## Data Availability

The datasets used and/or analyzed during the study are available from the corresponding author upon reasonable request.
