# Supplementary figures and images for: Anatomical changes of Tenebrio molitor and Tribolium castaneum during complete metamorphosis
Source: Cell Tissue Res. 2024 Feb 27;396(1):19–40. doi: 10.1007/s00441-024-03877-8 (PMC10997553; doi:10.1007/s00441-024-03877-8)

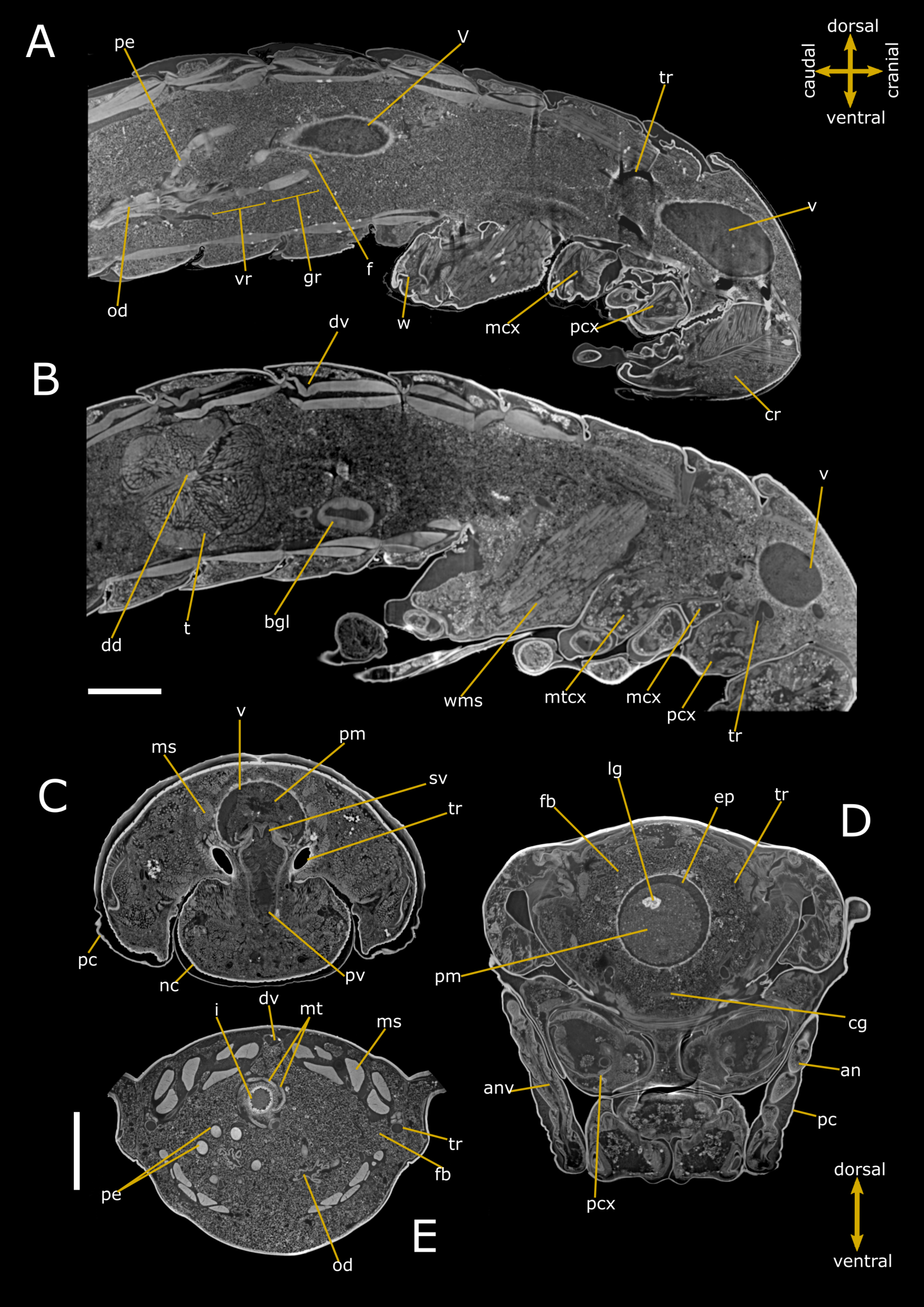

Supplement: Supplementary file 1 — Supplementary Fig. S1 Internal anatomy of Tenebrio molitor pupae. Two-dimensional PhC micro-CT-based longitudinal of female (A) and male (B) 8-day-old showing gonads, and cross sections at cranial (C; 8-day-old male), thoracic (D; 5-day-old female) and abdominal (E; 5-day-old female) level. bgl: bean-shaped accessory glands; an: antennomere; anv: antennal nerve; cg: connective of ganglia; cr: cerebrum; dd: deferent duct; dv: dorsal vessel; ep: ectoperitrophic space; f: apical filaments; fb: fat bodies; gr: germinarium; i: ileum; lg: larval gut; mcx: mesocoxa; ms: muscle; mt: malpighian tubules; mtcx: metacoxa; nc: new cuticle; od: oviduct; pc: pupal cuticle; pcx: procoxa; pe: pre-vitellogenic egg; pm: peritrophic matrix; pv: proventriculus; sv: stomodeal valve; t: testis; tr: tracheae; v: ventriculus; vr: vitellarium; wms: wing muscles. Scale bars:1 mm (A–E) (TIFF 15091 KB) [file 441_2024_3877_MOESM1_ESM.tiff]

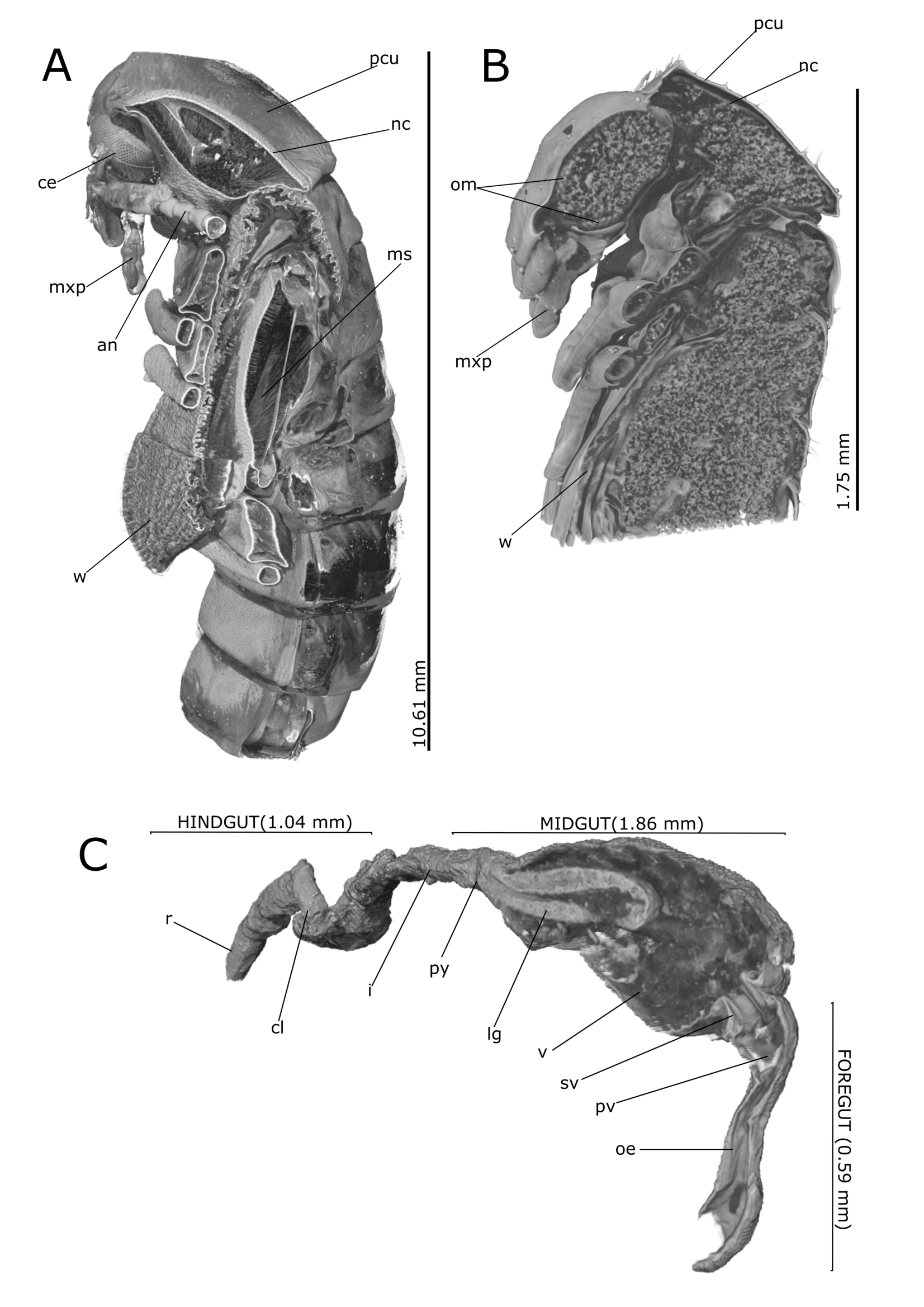

Supplement: Supplementary file 2 — Supplementary Fig. S2 3D volumetric rendering of Tenebrio molitor (A; 5-day-old female) and Tribolium castaneum (B; 5-day-old male) pupal stage, showing the newly formed cuticle (nc) enwrapped by the pupal cuticle (pc). (C) Segmented alimentary systems of Tribolium castaneum at late pupal stage (5-day-old female). an: antennomere; ce: compound eye; cl: colon; i: ileum; lg: larval gut; ms: muscle; mxp: maxillary palp; oe: oesophagus; om: ommatidia; pv: proventriculus; py: pyloric valve; r: rectum; sv: stomodeal valve; v: ventriculus; w: wing (TIFF 1757 KB) [file 441_2024_3877_MOESM2_ESM.tiff]

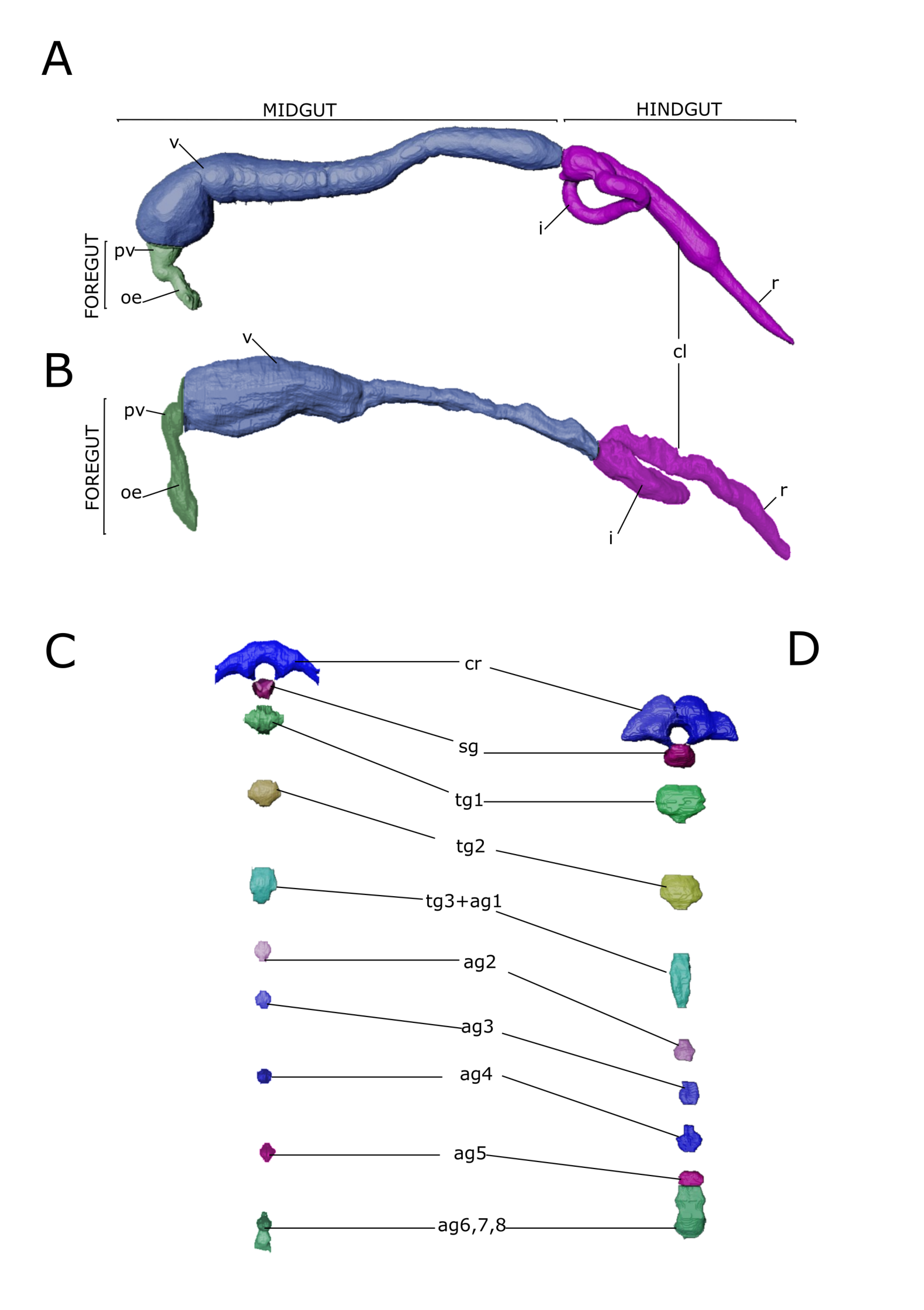

Supplement: Supplementary file 3 — Supplementary Fig. S3 3D reconstruction of the segmented pupal alimentary (A, B) and nervous systems (C, D) of Tenebrio molitor (A and C; 5-day-old) and Tribolium castaneum (B and D; 5-day-old) to obtain volumetric measurements as reported in Table 1 and 2. ag2-5: abdominal ganglia from 2 to 5; ag6, 7, 8: terminal abdominal ganglia fused to form a large caudal ganglion; cl: colon; cr: cerebrum; i: ileum; oe: oesophagus; pv: proventriculus; r: rectum; sg: subesophagean ganglion; tg1 and 2: thoracic ganglia 1 and 2; tg3 + ag1: complex of thoracic ganglion 3 and abdominal ganglion 1; v: ventriculus (TIFF 723 KB) [file 441_2024_3877_MOESM3_ESM.tiff]
